# Comparative Analysis of Systemic Inflammatory Biomarkers Across Multiple Antiseizure Medications: A Single-Center Retrospective Cohort Study of 1782 Patients

**DOI:** 10.3390/jcm14155190

**Published:** 2025-07-22

**Authors:** Kyung-Il Park, Sungeun Hwang, Hyoshin Son, Hyunah Yu, Jua Kim, Kon Chu, Ki-Young Jung, Sang Kun Lee

**Affiliations:** 1 Department of Neurology, Seoul National University Hospital Healthcare System Gangnam Center, Seoul 06236, Republic of Korea; ideopki@gmail.com; 2 Department of Neurology, Seoul National University College of Medicine, Seoul 03080, Republic of Korea; stemcell.snu@gmail.com (K.C.); jungky@gmail.com (K.-Y.J.); 3 Department of Neurology, Ewha Womans University Mokdong Hospital, Seoul 07985, Republic of Korea; neurosung@gmail.com; 4 Department of Neurology, Catholic University of Korea Eunpyeong St Mary’s Hospital, Seoul 03312, Republic of Korea; hson727@gmail.com; 5 Department of Neurology, Seoul National University Hospital, Seoul 03080, Republic of Korea; hacapjj04@snu.ac.kr (H.Y.); r4292r@snu.ac.kr (J.K.)

**Keywords:** antiseizure medication, inflammation markers, valproate, topiramate, carbamazepine

## Abstract

**Background/Objectives**: The aim of this study was to elucidate the associations between the use of various ASMs and systemic anti-inflammatory effects in a single large cohort using routine blood tests. **Methods**: Patients who underwent blood tests within three months of their first visit to our clinic were included. The systemic inflammatory index (SII, platelet × neutrophil/lymphocyte ratio), neutrophil–lymphocyte ratio (NLR), platelet–lymphocyte ratio (PLR), and fibrinogen–albumin ratio (FAR) were compared across specific ASMs. Data from a total of 1782 patients with epilepsy were analyzed. **Results**: Multiple linear regression analysis revealed that valproate use was significantly associated with lower SII, PLR, and FAR values. Additionally, carbamazepine and oxcarbazepine use were associated with the FAR, whereas topiramate use was associated with the PLR. When a dichotomized category for each inflammatory marker was used, dividing the lowest quartile and the other quartiles, VPA use was significantly associated with all four markers. Topiramate use was associated with lower SII, NLR, and PLR values, and carbamazepine use was associated with lower SII, FAR, and PLR values. **Conclusions**: These findings highlight the closer association between valproate, compared to other ASMs, and systemic inflammatory responses. These findings may offer valuable insights into the underlying mechanisms of the therapeutic effects of valproate.

## 1. Introduction

Neuro-inflammation has long been suggested to play a role in the processes of ictogenesis and epileptogenesis [1]. Brain-borne and systemic inflammation are linked to each other and increase the inflammatory cascade through the leaky blood–brain barrier, affecting epilepsy or recurrent seizures. Ample evidence from animal studies in which COX-2 blockers, aspirin, and nonsteroidal anti-inflammatory drugs were found to decrease spontaneous seizures substantiates the findings of clinical studies in which inflammatory molecules, high mobility group box-1 (HMGB-1), tumor necrosis factor (TNF)-α, the inflammasome, interleukin (IL)-1, and IL-6 are targeted.

Antiseizure medications (ASMs) have diverse mechanisms of action. We selected ASMs and their combination according to patients’ epilepsy syndromes and comorbidities. Additionally, we considered the pharmacodynamic mechanisms of ASMs initiated in polytherapy to minimize adverse events and maximize the synergistic effect. With respect to the various mechanisms of action of ASMs, some ASMs have been reported to have explicit direct anti-inflammatory mechanisms through inflammatory cytokine assays.

Simple inflammatory biomarkers, such as the systemic inflammatory index (SII), neutrophil–lymphocyte ratio (NLR), platelet–lymphocyte ratio (PLR), and fibrinogen–albumin ratio (PAR), are widely acknowledged because they are easy to calculate by combining routine blood test results at low cost and reflect the systemic inflammation status. The SII can be a prognostic marker of various non-neurological diseases, including gastric cancer [2], bladder tumors [3], coronary artery disease [4], and COVID-19 [5]. Since the SII was first introduced in 2014 [6], it has also been investigated in multiple neurological diseases, such as Parkinson’s disease [7], dementia [8], spinocerebellar ataxia [9], multiple sclerosis [10], autoimmune encephalitis [11], myasthenia gravis [12], Huntington’s disease [13], ischemic stroke [14,15], and brain tumors [16]. The SII is also correlated with chronic small vessel disease burden, explaining the associated inflammatory mechanism [17,18]. Additionally, in the epilepsy field, the SII and NLR have been suggested as biomarkers that can differentiate between true seizures and psychogenic nonepileptic seizures [19].

In contrast to previous studies investigating the prognostic value of inflammatory biomarkers, we attempted to utilize these markers to assess the effect of ASMs on the systemic inflammatory status.

## 2. Materials and Methods

### 2.1. Study Population

The retrospective cohort included patients with epilepsy, with a minimum follow-up duration of three years. This cohort included all patients who had their first visit to our clinic between 2008 and 2017. Data were collected from the Seoul National University Hospital Adult Epilepsy Registry in the Era of Newer Antiseizure Drug Exposure (SERENADE) study database. The detailed design of this retrospective cohort was previously described [20].

In this population, data from routine blood tests performed within three months of the first clinic visit were ultimately analyzed. The patients with acute infections, inflammatory comorbidities, or autoimmune encephalitis were excluded. Electronic hospital records were thoroughly reviewed to extract information regarding sex, age at epilepsy onset, age at blood sampling, number of seizures in the past 3 months, epilepsy classification, MRI findings, and ASM status and types at the time of blood sampling. From the routine blood test data, the SII, which is calculated as platelet × neutrophil/lymphocyte, NLR, PLR, and FAR were calculated and compared across ASM categories, which included levetiracetam (LEV), valproate (VPA), lamotrigine (LTG), topiramate (TPM), oxcarbazepine (OXC), and carbamazepine (CBZ).

### 2.2. Statistical Analysis

Continuous variables are presented as the means ± standard deviations. Univariate analyses, including independent *t* tests, chi-square tests, and correlation analyses, were conducted for each variable. Variables with significant *p*-values (<0.05) and clinically relevant variables were subsequently incorporated into multiple linear regression models. For each ASM, exposure was defined based on whether the patient was taking the medication, regardless of whether it was used as monotherapy or as part of polytherapy. To adjust for potential confounding, treatment type (monotherapy vs. polytherapy) was included as a covariate in all multivariable regression models. For binary logistic regression analysis, each inflammatory marker was dichotomized into the lowest quartile and the remaining higher quartiles to identify odds ratios. SPSS (version 25, IBM, Chicago, IL, USA) was used for statistical analysis, and GraphPad Prism (version 9, Dotmatics, San Diego, CA, USA) was used for graph generation.

## 3. Results

Overall, blood data from 1782 patients (female = 814, 45.7%) were analyzed. The cohort consisted of patients with focal epilepsy (77.8%), generalized epilepsy (15.4%), and combined types (5.5%). The etiologies of epilepsy included structural (626, 35.1%), genetic (253, 14.2%), immune (84, 4.7%), infectious (51, 2.9%), hypoxic (5, 0.3%), metabolic (3, 0.2%), and unknown (760, 42.6%) causes. The MRI findings were classified as normal (50.8%), cerebromalacia (11.4%), hippocampal sclerosis (6.5%), or vascular anomaly (4.9%) in order of frequency. The mean epilepsy onset age was 31.0 ± 20.2 years, and the mean age at blood sampling was 37.5 ± 18.0 years (range: 11–91 years).

A total of 838 patients (47.0%) were drug naïve. Among the remaining patients, 515 were on one ASM, 252 on two, 119 on three, 41 on four, 13 on five, and three on more than four ASMs. The percentages of patients using specific ASMs were as follows: LEV (387, 21.7%), VPA (297, 16.7%), OXC (189, 10.6%), TPM (188, 10.5%), LTG (173, 9.7%), CBZ (155, 8.7%), phenytoin (80, 4.5%), zonisamide (54, 3.0%), clobazam (37, 2.1%), pregabalin (28, 1.6%), gabapentin (9, 0.5%), phenobarbital (14, 0.8%), vigabatrin (6, 0.3%), and lacosamide (12, 0.7%).

Initially, clinical parameters were compared between ASM users and nonusers at the time of blood sampling. ASM users exhibited an earlier epilepsy onset (24.9 ± 17.0 vs. 37.9 ± 21.3 years, *p* < 0.001), younger age at blood sampling (34.0 ± 15.4 vs. 41.4 ± 19.8 years, *p* < 0.001), longer epilepsy duration (8.9 ± 10.5 vs. 3.4 ± 7.2 years, *p* < 0.001), more seizures in the past three months (10.1 ± 22.3 vs. 6.8 ± 19.5, *p* = 0.02), and a greater proportion of febrile seizures (9.9% vs. 5.4%, *p* = 0.001). MRI lesions were more common in ASM users (52.3% vs. 46.0%, *p* = 0.015), and the interval from the last seizure to blood sampling was longer in ASM users than in drug-naïve patients (*p* < 0.001). The SII was significantly lower in ASM users (511.6 ± 591.9 vs. 687.2 ± 901.1, *p* < 0.001). Comparative data between ASM users and drug-naïve patients are summarized in Table 1.

Multiple linear regression was subsequently performed to identify independent factors influencing inflammatory marker levels. VPA emerged as the only ASM significantly associated with the SII (*p* = 0.026), whereas TPM, LTG, and CBZ were not significantly associated with the SII despite their initial associations in the univariate analysis. The mean SII values for VPA users and nonusers were 430.8 ± 419.7 and 626.8 ± 805.0, respectively. Similar analyses were performed for the PLR, FAR, and NLR. The use of VPA was significantly associated with the FAR (*p* = 0.01) and PLR (*p* < 0.001). The PLRs for VPA users and nonusers were 727.4 ± 474.7 and 954.0 ± 861.3, respectively, and the FARs were 57.1 ± 24.6 and 66.6 ± 23.5, respectively. A lower FAR was associated with CBZ (*p* = 0.002) and OXC (*p* = 0.018) use, whereas no significant associations were observed for the other markers. TPM use was associated with the PLR (*p* = 0.008). The results of standardized β coefficients and corresponding *p*-values are presented in Table 2.

**Figure 1:**
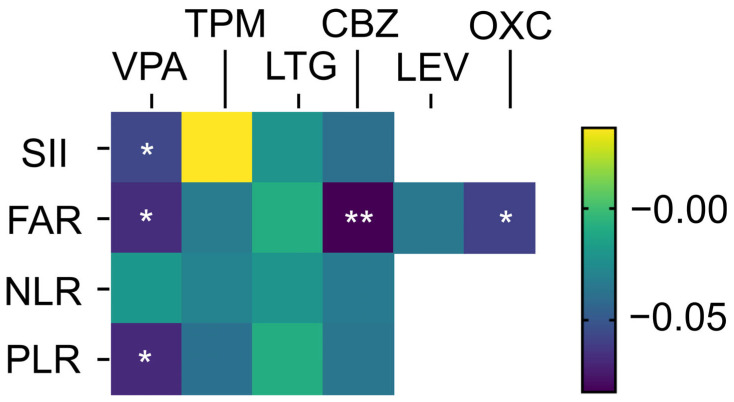
Linear regression-based anti-inflammatory profiles of antiseizure medications (ASMs). Heatmap showing standardized beta coefficients from multiple linear regression analyses, in which systemic inflammatory markers were treated as continuous dependent variables. These results demonstrate the distinct anti-inflammatory effects of commonly used ASMs. * *p* < 0.05; ** *p* < 0.01; VPA, valproate; TPM, topiramate; LTG, lamotrigine; CBZ, carbamazepine; LEV, levetiracetam; OXC, oxcarbazepine.

**Figure 2:**
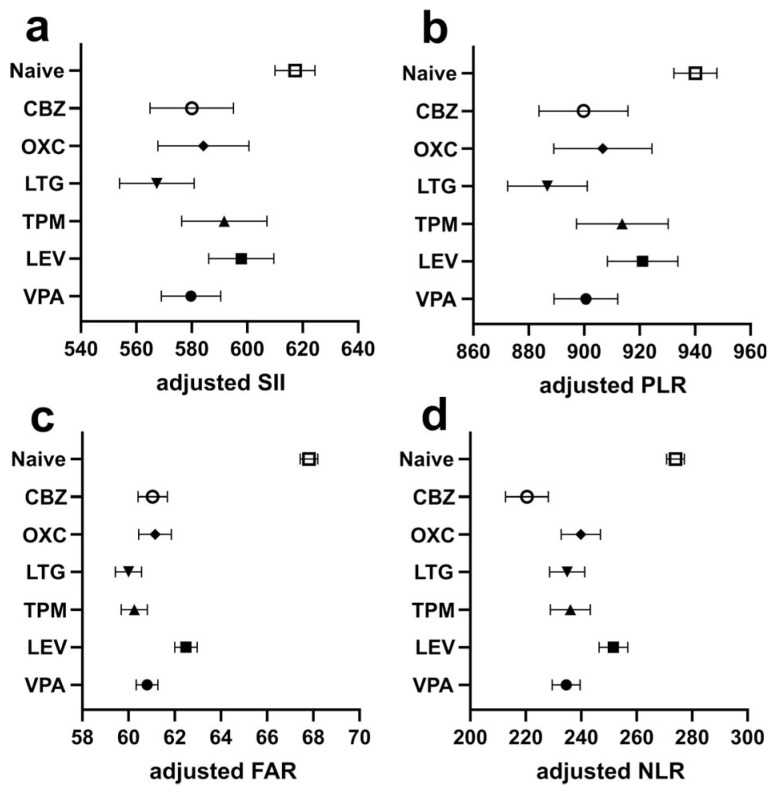
Adjusted levels of systemic inflammatory markers across antiseizure medication groups. Adjusted mean values with standardized errors of the means for each inflammatory marker are shown according to the ASM exposure status. Analyses were based on multiple linear regression, adjusting for potential confounders. (**a**) Systemic inflammation index (SII), (**b**) platelet–lymphocyte ratio (PLR), (**c**) fibrinogen–albumin ratio (FAR), and (**d**) neutrophil–lymphocyte ratio (NLR).

Statistical associations between the use of ASMs and inflammatory markers are visualized in a heatmap in Figure 1, and the adjusted values for each marker across the ASMs are displayed in panels Figure 2a–d.

**Figure 3:**
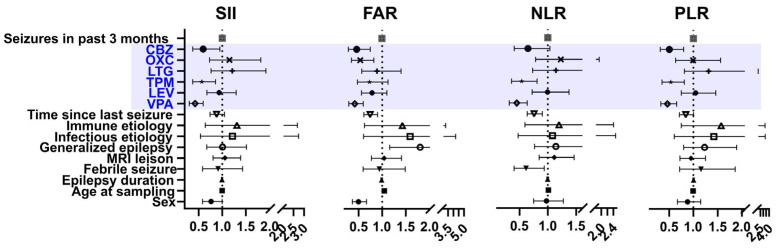
Forest plots showing the associations between systemic inflammatory indices and clinical parameters, including the antiseizure medications used. Each marker, SII, FAR, NLR, and PLR, was dichotomized into the lowest quartile and the remaining higher quartiles. Odds ratios with 95% confidence intervals are presented, with the lowest quartile used as the reference. Valproate had the lowest odds ratios across multiple markers. Topiramate, carbamazepine, and oxcarbazepine showed selective associations, whereas levetiracetam and lamotrigine generally showed no significant differences.

**Figure 4:**
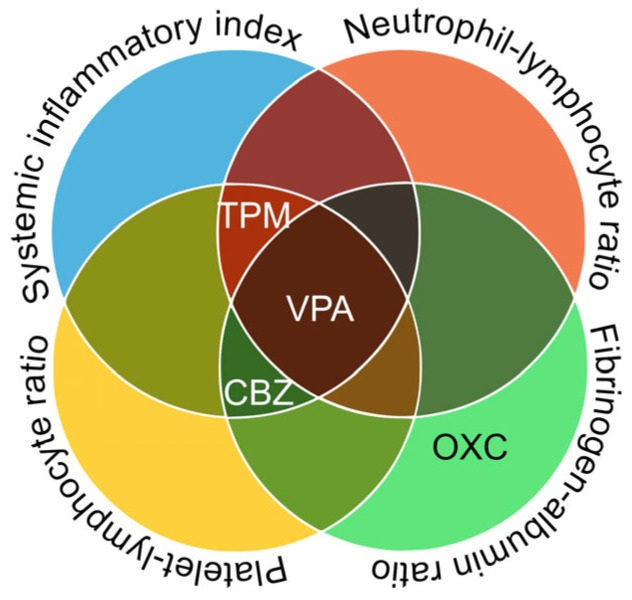
Overlap of significant associations between ASMs and inflammatory markers according to logistic regression. Venn diagram illustrating the overlap of ASMs significantly associated with dichotomized inflammatory markers, based on binary logistic regression. Valproate was consistently associated with all four markers. Topiramate and carbamazepine were each associated with three markers, whereas oxcarbazepine was associated with one.

Next, using dichotomized categories of the lowest quartile versus all other quartiles, we performed binary logistic regression analyses. Continuous inflammatory biomarkers were categorized into binary variables to improve interpretability and allow for approximate comparisons of ASM-related effects using odds ratios. This approach was intended to support visual clarity and highlight potential differences in biomarker levels across groups. Low SII and FAR values were independently associated with the use of VPA, TPM, and CBZ. The NLR was associated with the use of VPA and TPM, whereas the PLR was associated with the use of VPA, TPM, and CBZ (Figure 3). The corresponding odds ratios are presented as forest plots in Figure 4.

To evaluate the robustness of the findings, sensitivity analyses were conducted using tertile-based cutoffs (Tables S1–S4), which showed similar results. We also performed separate analysis restricted to monotherapy users. Binary logistic analysis based on the use versus nonuse of each ASM showed that VPA and LEV are associated with the SII and PLR (Tables S5–S9). In contrast, multiple linear regression found that only VPA was associated with the PLR, while no other ASMs showed such association (Table S10).

## 4. Discussion

In this study, we demonstrated that several ASMs were associated with lower inflammatory markers, with distinct patterns observed among individual medications. In the multivariate analysis of continuous variables, VPA was significantly associated with three of the four inflammatory markers (SII, PLR, and FAR), whereas CBZ, TPM, and OXC were each associated with only one marker. The dichotomized analysis provided further clarity. VPA was significantly associated with all four markers, whereas TPM and CBZ were associated with three other markers. In contrast, no significant associations were observed for LEV or LTG in either analysis. These findings suggest that the extent of inflammatory marker associations with ASMs varies across ASMs. This study is the first to comprehensively investigate the association between various inflammatory markers and various ASMs in a single cohort. Previous research has demonstrated the potential anti-inflammatory effects of ASMs, primarily through cytokine-based evidence, but these studies had a relatively small number of participants.

Among the ASMs we evaluated in our study, VPA use was consistently associated with lower values for the SII, PLR, and FAR than nonuse of VPA. Findings from previous animal studies support our findings, revealing that VPA reduces localized edema and body temperature, resembling the effects of NSAIDs [21]. In an animal model of neuropathic pain, two weeks of VPA treatment increased the threshold of paw withdrawal latency and decreased the levels of inflammatory mediators, including phosphorylated NF-κB, which is a common transcription factor that mediates the production of pro-inflammatory cytokines, inducible nitric oxide synthetase, COX-2, TNF-α, and IL-1β [22]. A similar result was directly found in an in vitro study, where VPA reduced the levels of pro-inflammatory cytokines, such as IL-6 and TNF-α, whereas CBZ, phenobarbital, and phenytoin did not [23]. Furthermore, VPA downregulated IL-6 expression in spinal dorsal horns, and recombinant IL-6 administration reversed this effect, suggesting that the IL-6 pathway is involved in the mechanism of action of VPA [24]. 

These findings align with our results, suggesting that the anti-inflammatory mechanisms of VPA are mediated through the modulation of cytokine pathways. A clinical study of 21 generalized epilepsy patients revealed that VPA treatment for 4–6 months decreased the serum level of IL-6 but not that of IL-1β or TNF-α [25].

However, compelling results exist. For example, a prospective clinical study in which neither VPA nor LEV treatment for 3 months changed the levels of IL-1β, IL-6, TNF-α, and MCP-1 in the peripheral blood revealed that both ASMs merely changed the number of CD3 + CD4+ lymphocytes [26]. The critical limitation of this study, however, is its small sample size of only 36 patients. Another study revealed a positive association between IL-6 and VPA use in healthy volunteers [27]. The difference in findings may be attributable to differences in the study samples.

One notable finding of this study is the comparison of various ASMs regarding inflammatory markers in epilepsy patients. Although univariate analysis revealed that four ASMs, including VPA, LTG, CBZ, and TPM, were significantly associated with the SII, NLR, PLR, and FAR, only VPA remained statistically significant according to multivariate analysis of three parameters, and CBZ and OXC were significantly associated with one parameter each. Previously, in a pilocarpine-induced status epilepticus model, compared with VPA, LEV, and CBZ, LEV suppressed microglial activation and spontaneous recurrent seizures; VPA suppressed neuro-inflammation, but CBZ did not have antiseizure or anti-inflammatory effects. LEV and VPA decreased the mRNA levels of IL-1β and TNF-α in the hippocampus [28]. In contrast, one study using different animal seizure models and comparing the effects of CBZ and VPA on cytokine expression revealed that CBZ reduced IL-1β and TNF-α expression, but VPA did not [29].

In an in vitro study of peripheral blood mononuclear cells, CBZ decreased the levels of pro-inflammatory cytokines such as IL-2 and IL-4 but increased the levels of the anti-inflammatory cytokine IL-10 [30]. CBZ treatment before surgery decreased pain sensation and the IL-6 level in a clinical study, suggesting that anti-inflammation is one of the mechanisms underlying postoperative pain control by CBZ [31]. Another study supported this result, showing a dose-dependent reduction in edema in rat paws caused by CBZ [32]. Compared with CBZ use, VPA use led to lower levels of IL-2, IL-8, and TNF-α in 120 epilepsy patients, including 60 VPA- and 60 CBZ-treated patients [33], which is similar to our findings.

Also consistent with our study findings, TPM use dose-dependently reduced the levels of inflammatory-related molecules, such as IL-1β, IL-6, and NF-kB, in the rat brain [34]. This finding is also supported by findings from a study on rat microglial cell culture. IL-1β and IL-6 release by lipopolysaccharide induction was decreased by TPM [35]. However, evidence on systemic levels of systemic markers, such as those in our study, is scarce.

Our study revealed no association between inflammatory markers and LTG. A previous study involving 145 poststroke epilepsy patients compared a LTG plus VPA treatment group and a VPA-only group in terms of the serum HMGB-1, matrix metalloproteinase (MMP)-9, and IL-6 levels. Both groups presented a reduction in these markers after treatment, and those of the LTG plus VPA group were significantly lower than those of the VPA-only control group. However, this result does not necessarily indicate that LTG has a pure anti-inflammatory effect because the dual therapy group had much less epileptiform discharge after treatment [36].

The data concerning the anti-inflammatory effects of LEV are somewhat controversial. LEV treatment at a concentration of 50 µg/mL, which mimics the serum concentration, was used in an astrocyte–microglia coculture model, and the addition of lipopolysaccharide and IL-1β to induce an inflammatory condition reversed the electrophysiological properties to a non-inflammatory level. The same study group reported that LEV alleviated the resting membrane potential and that its action was mediated by TGF (transforming growth factor)-β1 [37], suggesting that one of the antiseizure mechanisms of LEV is anti-inflammatory. VPA led to decreased COX-2, prostaglandin E2, NF-kB, and TNF-α expression in the brain after 30 days of treatment in a rat model of chemotherapy-induced memory impairment [38]. LEV reduced the infiltration of systemic inflammatory cells [39] and microglial activation and decreased the serum levels of TNF-α, IL-1β, and IL-6 [40]. However, the anti-inflammatory effect of LEV was not evident in an animal autoimmune encephalomyelitis model, where the levels of IL-1β, IL-10, and TGF-β in the spinal cord and spleen did not significantly differ between LEV-treated and control animals [41]. Compared with that in the VPA-only control group, the effect of LEV addition on VPA in the pediatric epilepsy population was investigated. Both groups exhibited decreased CCL2 levels, but serum IL-1β levels were not altered [42]. LEV had direct effects on IL-1β in a previous clinical study of 22 epilepsy patients, which revealed significantly decreased IL-1β levels two hours after LEV administration [43]. We found an association between LEV usage and inflammatory marker levels in this study. The contradictory results might be due to the different models, species, or concentrations used in each experiment and patient.

In contrast to the accumulating data regarding ASMs and anti-inflammatory molecules, the underlying mechanism of the anti-inflammatory effect of specific ASMs is not well established. Biomarkers such as the SII, NLR, PLR, and FAR reflect complex immune interactions [44,45]. The SII, NLR, and PLR reflect the roles of neutrophils, lymphocytes, and platelets in inflammation. Platelets are blood components associated with innate immunity [46], whereas lymphocytes are linked to adaptive immunity [18]. Neutrophils secrete various inflammatory cytokines, contributing to innate immune responses.

The FAR also reflects the inflammatory process of fibrinogen and additionally reflects nutritional status via albumin levels in cancer [47], heart failure [48], diabetic neuropathy [49], and stroke [50]. Fibrinogen is a marker of inflammation because its levels are increased in response to inflammation [20]. Fibrinogen has the potential to bind platelets [50] as an acute-phase protein. Additionally, albumin has anti-inflammatory properties, inhibits platelet aggregation, and reduces oxidative stress [50]. In line with this, low albumin is suggested as a poor prognostic factor in Guillain–Barré syndrome [51], and high albumin indicates a good prognosis of autoimmune encephalitis [52]. All these markers are influenced by innate and adaptive immunity and have been utilized in diverse clinical contexts to assess systemic inflammation.

Although the exact mechanism of the anti-inflammatory action of VPA remains unclear, a study on a hepatic cell line demonstrated that VPA at a clinically relevant dose decreased the levels of TGF-β1, a pivotal component in blood–brain barrier-related neuro-inflammation and subsequent astrogliosis activation [53]. Another plausible explanation is that VPA may suppress inflammatory processes through platelet modulation, as we observed a decrease in the PLR, but not in the NLR, when analyzed as a continuous variable via linear regression. This finding is consistent with the role of platelets as a source of pro-inflammatory cytokines, given their ability to activate NF-kB [54], which, in turn, induces cytokine production and activates innate immune responses. This assumption is further supported by the known adverse effects of VPA on thrombocytopenia. Platelets aggregate with leukocytes via platelet–leukocyte receptors [55], subsequently activating innate immunity by responding to acute vascular injury signals. Given that a PLR increase is evident in vascular disease, this concept can be adopted in epilepsy because epilepsy is a condition of elevated inflammatory status caused by recurrent seizures. Platelet-induced leukocyte adhesion to the endothelium and the associated increase in cytokine release could contribute to neuro-inflammation. Neutrophils further activate macrophages through the formation of neutrophil extracellular traps, leading to the activation of the NLRP3 inflammasome. NLRP3, in turn, triggers the production of the key pro-inflammatory cytokine IL-1β [56,57]. Additionally, platelets contain COX enzymes that synthesize prostanoids, further propagating the inflammatory cascade. However, the reason why VPA usage does not impact the NLR remains unclear. A prospective study in which clinical variables are controlled for is warranted to clarify this differential association and provide a clearer understanding of the mechanisms involved.

VPA may exert anti-inflammatory effects by modulating key molecular mediators implicated in seizure-related neuro-inflammation. Among these, HMGB1 is a well-established damage-associated molecular pattern molecule that triggers downstream inflammatory cascades. Increased HMGB1 levels have been associated with microglial activation, cytokine release, and enhanced neuronal excitability. Moreover, HMGB1-driven inflammation contributes to the disruption of the blood–brain barrier (BBB), allowing peripheral immune cells and serum proteins, such as albumin, to infiltrate brain tissue and exacerbate neuro-inflammation via pathways such as TGF-β. VPA has been associated with reduced HMGB1 levels in patients with post-stroke epilepsy [36], suggesting that its anti-inflammatory action may lead to secondary stabilization of the BBB by attenuating HMGB1-mediated permeability changes. In addition, VPA has been linked to lower expression of MMP-9, a proteolytic enzyme directly involved in degrading tight junction proteins and compromising BBB integrity. Experimental studies have shown that the HMGB1 monoclonal antibody can suppress MMP-9 expression and prevent BBB leakage in animal models of status epilepticus [58]. Therefore, by reducing HMGB1-related inflammatory signaling, VPA may help to preserve BBB function.

Furthermore, VPA has been shown to act on mitogen-activated protein kinase (MAPK) pathways [59]. Activation of the MAPK cascade modulates the function of ion channels and neurotransmitter receptors, leading to increased neuronal excitability [60]. This heightened excitability, in turn, promotes the release of pro-inflammatory mediators and activation of glial cells, thereby contributing to the development of neuro-inflammation. By inhibiting MAPK signaling, VPA may suppress this excitability-driven inflammatory cascade, providing an additional mechanism by which it reduces seizure-associated neuro-inflammation.

Established evidence indicates that the JAK–STAT pathway is associated with seizure severity [61] and that STAT3 activation contributes to neuro-inflammation [62]. Experimental studies using spinal cord injury models have shown that valproate attenuates inflammation by inhibiting STAT1 signaling, suggesting that suppression of this pathway may underlie, at least in part, the anti-inflammatory effects of VPA.

Another potential mediator of the anti-inflammatory action of VPA is histone deacetylase (HDAC). A previous clinical study demonstrated that VPA reduces the risk of ischemic stroke through HDAC inhibition [63], and similar findings were reported in an in vivo stroke model [64]. Since HDACs are associated with inflammatory mediators, the ability of VPA to prevent stroke may be due to its ability to suppress inflammation. Previous studies have shown that ischemic stroke outcomes after intervention are associated with inflammatory markers similar to those evaluated in our study. Considering that the HDAC inhibition effect of VPA is linked to stroke outcomes through the modulation of inflammation, the decreases in inflammatory markers associated with VPA use in our study may support the anti-inflammatory effects of VPA and suggest that HDACs act as mediators of this process. Additionally, VPA has been shown to inhibit atherosclerosis, which is also associated with inflammation. A recent genome-wide association study further confirmed the association between VPA use and a reduced risk of stroke recurrence [65]. Proposed inflammatory mediators of ASMs are summarized in Table 3.

There are several limitations in this study. First, this retrospective cohort included new patients visiting our clinic to ensure the homogeneity in data quality. However, this approach limits the generalizability of ASM usage and may lead to the underrepresentation of older-generation ASMs. Second, because of the cross-sectional design, only a single time point is captured, which limits the ability to draw conclusions about causality or observe temporal changes. Dynamic changes in seizure status or general health conditions may influence the observed associations. Third, we did not perform inflammatory cytokine assays (e.g., IL-6, TNF-α) to directly support the proposed associations, which restricts our ability to explain the distinct contributions of innate and adaptive immunity. Nevertheless, the simplicity and accessibility of the inflammatory markers evaluated in this study may be considered strengths, particularly in retrospective clinical research involving a heterogeneous population. Fourth, the effects of ASMs on inflammation are not solely reflected by blood inflammatory markers because seizure burden may be a confounding factor. Given the reciprocal relationship between neuro-inflammation and seizures, where seizures themselves act as damage-associated molecular signals that can trigger or worsen inflammation, separating the direct anti-inflammatory effects of ASMs from their seizure-suppressing actions is difficult. However, in our study, ASM users had significantly higher seizure frequencies than did drug-naïve patients, and multivariate analysis revealed a significant association between inflammatory marker levels and ASM use. These findings suggest that the observed reduction in inflammatory marker levels is unlikely to be explained solely by seizure control. Fifth, potential bias may exist because of unmeasured confounders, such as concurrent medications, such as anti-inflammatory drugs, duration of treatment, or serum levels of ASMs. Further research is warranted to address these limitations. Sixth, although we identified statistical associations between certain inflammatory markers and specific ASMs, such as valproate, carbamazepine, oxcarbazepine, or topiramate, the clinical interpretation of these findings should be approached with caution. The standardized beta coefficients were relatively small compared to those observed with clinical factors whose relevance is more intuitively understood, such as infectious etiology or recent seizures. While our results are primarily statistical in nature, the differential inflammatory profiles observed across ASMs may reflect how each agent deploys distinct pleiotropic mechanisms. Such comparisons could help inform more refined therapeutic approaches for epilepsy and other inflammation-related conditions.

## 5. Conclusions

We found that, compared with the use of other antiseizure medications, VPA use was significantly associated with lower levels of systemic inflammatory markers. To our knowledge, this is the first study in which routine inflammatory marker levels were evaluated across multiple ASMs within a single relatively large dataset. Given that neuro-inflammation in epilepsy may be amplified by systemic inflammatory signaling, our findings suggest that VPA may exert a stronger anti-inflammatory effect than other ASMs, with potential implications for both epilepsy and neurodegenerative diseases. Further research into the immunomodulatory mechanisms of ASMs is needed to improve our understanding and explore opportunities for drug repurposing.

## Figures and Tables

**Table 1:** Comparison between patients receiving antiseizure medications and drug-naïve controls.

	Drug-Naïve	^5^ ASM User	*p* Value
Sex (female–male)	383:455	431:513	1.000
Age at epilepsy onset	37.9 ± 21.3	24.9 ± 17.0	<0.001
Age at sampling	41.4 ± 19.8	34.0 ± 15.4	<0.001
Epilepsy duration	3.4 ± 7.2	8.9 ± 10.5	<0.001
Seizure frequency in the past 3 months	6.8 ± 19.5	10.1 ± 22.3	0.02
Seizure classification			
Generalized	661 (78.9%)	726 (76.9%)	0.496
Focal	135 (16.1%)	140 (14.8%)	0.346
Epilepsy etiology			0.629
Genetic	117 (14.0%)	136 (14.4%)	
Hypoxic	1 (0.1%)	4 (0.4%)	
Immune	44 (5.3%)	40 (4.2%)	
Infectious	27 (3.2%)	24 (2.5%)	
Metabolic	2 (0.2%)	1 (0.1%)	
Structural	285 (34.0%)	341 (36.1%)	
Unknown	362 (43.2%)	398 (42.2%)	
History of febrile seizures	45 (5.4%)	93 (9.9%)	0.001
MRI lesion (+)	347 (46.0%)	417 (52.3%)	0.015
Time since last seizure			<0.001
~1 week	379 (46.7%)	275 (29.3%)	
1 week~1 month	245 (30.2%)	365 (39.0%)	
≥1 month	188 (23.2%)	297 (31.7%)	
^1^ SII	687.2 ± 901.1	511.6 ± 591.9	<0.001
^2^ PLR	1016.0 ± 956.4	827.8 ± 650.8	<0.001
^3^ NLR	297.6 ± 392.5	215.6 ± 206.3	<0.001
^4^ FAR	69.9 ± 25.9	60.6 ± 20.0	<0.001

^1^ SII, systemic inflammatory index; ^2^ PLR, platelet–lymphocyte ratio; ^3^ NLR, neutrophil–lymphocyte ratio; ^4^ FAR, fibrinogen–albumin ratio; ^5^ ASM, antiseizure medication.

**Table 2:** Multiple linear regression models for each inflammatory index.

	Standardized β Coefficient (*p* Value)			
	SII ^1^	PLR ^2^	NLR ^3^	FAR ^4^
Sex	-	-	-	−0.065 (0.013)
Epilepsy duration	−0.044 (0.147)	−0.039 (0.19)	−0.043 (0.118)	−0.063 (0.030)
Number of seizures in the past 3 months	−0.076 (0.007)	−0.08 (0.002)	−0.086 (0.001)	−0.043 (0.114)
Onset age	-	0.009 (0.76)	-	-
Febrile seizures	−0.042 (0.125)	−0.039 (0.124)	−0.043 (0.085)	−0.012 (0.634)
MRI lesion (+)	0.036 (0.201)	-	-	0.078 (0.004)
Age at sampling	−0.001 (0.983)	-	0.061 (0.021)	0.329 (<0.001)
Time since last seizure	−0.17 (<0.001)	−0.172 (<0.001)	−0.215 (<0.001)	−0.117 (<0.001)
Generalized epilepsy	−0.021 (0.468)	-	−0.031 (0.241)	0.158 (0.022)
Infectious etiology	0.163 (<0.001)	0.157 (<0.001)	0.155 (<0.001)	0.183 (<0.001)
Immune etiology	0.076 (0.005)	0.077 (0.002)	0.051 (0.041)	0.031 (0.235)
Structural etiology	-	0.008 (0.767)	-	-
Genetic etiology	-	-	-	−0.083 (0.237)
Valproate	−0.057 (0.048)	−0.068 (0.016)	−0.02 (0.475)	−0.067 (0.025)
Topiramate	−0.036 (0.246)	−0.038 (0.191)	−0.03 (0.287)	−0.033 (0.279)
Lamotrigine	−0.021 (0.476)	−0.009 (0.749)	−0.021 (0.436)	−0.009 (0.745)
Carbamazepine	−0.039 (0.181)	−0.036 (0.185)	−0.034 (0.199)	−0.082 (0.005)
Oxcarbazepine	-	-	-	−0.059 (0.042)
Levetiracetam	-	-	-	−0.035 (0.258)
Polytheraphy	−0.016 (0.677)	−0.006 (0.860)	−0.021 (0.540)	−0.014 (0.762)

^1^ SII, systemic inflammatory index; ^2^ PLR, platelet–lymphocyte ratio; ^3^ NLR, neutrophil–lymphocyte ratio; ^4^ FAR, fibrinogen–albumin ratio.

**Table 3:** Inflammatory mediators of antiseizure medications.

Antiseizure Medications	Mediators
Valproate	^1^ HMGB-1, ^2^ MMP-9, ^3^ IL-6, ^4^ TGF-β1, ^5^ NF-κb, ^6^ NLRP3, ^7^ JAK/STAT, ^8^ HDAC
Carbamazepine	IL-1β, IL-2, IL-4, ^9^ TNF-α
Topiramate	IL-1β, IL-6, NF-κb
Levetiracetam	TGF-β, ^10^ COX-2, NF-κb, TNF-α, IL-1β

^1^ HMGB, high mobility group box; ^2^ MMP, matrix metalloproteinase; ^3^ IL, interleukin; ^4^ TGF, transforming growth factor; ^5^ NF, nuclear factor; ^6^ NLRP, NOD-like receptor Pyrin domain; ^7^ JAK/STAT, Janus Kinase/Signal Transducer and Activator of Transcription; ^8^ HDAC, histone deacetylase; ^9^ TNF, tumor necrosis factor; ^10^ COX, cyclooxygenase.

## Data Availability

The datasets used and analyzed during the current study are available from the corresponding author upon reasonable request in anonymized form.

## Supplementary Materials

The following supporting information can be downloaded at https://www.mdpi.com/article/10.3390/jcm14155190/s1: Table S1. Associations between systemic inflammatory index and clinical parameters, including the antiseizure medications. Binary logistic regression was performed using tertile-based dichotomization. The dependent variable was dichotomized into the lowest tertile versus the combined higher tertiles. Asterisks indicate p values lower than 0.05; Table S2. Associations between fibrinogen-albumin ratio and clinical parameters, including the antiseizure medications. Binary logistic regression was performed using tertile-based dichotomization. The dependent variable was dichotomized into the lowest tertile versus the combined higher tertiles; Table S3. Associations between neutrophil-lymphocyte ratio and clinical parameters, including the antiseizure medications. Binary logistic regression was performed using tertile-based dichotomization. The dependent variable was dichotomized into the lowest tertile versus the combined higher tertiles; Table S4. Associations between platelet-albumin ratio and clinical parameters, including the antiseizure medications. Binary logistic regression was performed using tertile-based dichotomization. The dependent variable was dichotomized into the lowest tertile versus the combined higher tertiles; Table S5. Binary logistic regression analysis of valproate monotherapy user; Table S6. Binary logistic regression analysis of topiramate monotherapy user; Table S7. Binary logistic regression analysis of carbamazepine monotherapy user; Table S8. Binary logistic regression analysis of lamotrigine monotherapy user; Table S9. Binary logistic regression analysis of levetiracetam monotherapy user; Table S10. Multiple linear regression models for each inflammatory index in monotherapy population.

## Abbreviations

The following abbreviations are used in this manuscript:

ASM	antiseizure medication
SII	systemic inflammatory index
NLR	neutrophil–lymphocyte ratio
PLR	platelet–lymphocyte ratio
FAR	fibrinogen–albumin ratio
COX	cyclooxygenase
HMGB-1	high mobility group box-1
TNF	tumor necrosis factor
IL	interleukin
LEV	levetiracetam
VPA	valproate
LTG	lamotrigine
TPM	topiramate
OXC	oxcarbazepine
CBZ	carbamazepine
BBB	blood–brain barrier
TGF	transforming growth factor
MMP	matrix metalloproteinase
MAPK	mitogen-activated protein kinase
HDAC	histone deacetylase
