# Supplementary material for: Comparative Analysis of Systemic Inflammatory Biomarkers Across Multiple Antiseizure Medications: A Single-Center Retrospective Cohort Study of 1782 Patients
Source: J Clin Med. 2025 Jul 22;14(15):5190. doi: 10.3390/jcm14155190 (PMC12347507; doi:10.3390/jcm14155190)
Supplement: Supplementary file 1 [file jcm-14-05190-s001.zip › jcm-3749150-supplementary.pdf]

Table S1. Associations between systemic inflammatory index and clinical parameters, including the antiseizure medications. Binary logistic regression was performed using tertile-based dichotomization. The dependent variable was dichotomized into the lowest tertile versus the combined higher tertiles. Asterisks indicate p values lower than 0.05.

	B	Standard error of coefficient	P value	Odds ratio
Sex	0.182	0.116	0.116	1.200
Age at sampling	-0.002	0.003	0.657	0.998
Epilepsy duration	-0.004	0.007	0.533	0.996
Febrile seizures	0.065	0.205	0.752	1.067
MRI lesion (+)	0.191	0.119	0.107	1.210
Generalized epilepsy	-0.263	0.187	0.161	0.769
Infectious etiology	-0.504	0.380	0.185	0.604
Immune etiology	-0.590	0.294	**0.045***	0.555
Time since last seizure	0.343	0.072	**0.000***	1.409
Antiseizure medications				
Valproate	0.774	0.153	**0.000***	2.167
Levetiracetam	0.107	0.140	0.445	1.112
Topiramate	0.593	0.183	**0.001***	1.809
Lamotrigine	-0.063	0.198	0.749	0.939
Oxcarbazepine	-0.121	0.192	0.529	0.886
Carbamazepine	0.326	0.209	0.118	1.386

Table S2. Associations between fibrinogen-albumin ratio and clinical parameters, including the antiseizure medications. Binary logistic regression was performed using tertile-based dichotomization. The dependent variable was dichotomized into the lowest tertile versus the combined higher tertiles.

	B	Standard error of coefficient	P value	Odds ratio
Sex	0.648	0.133	**0.000***	1.911
Age at sampling	-0.037	0.005	**0.000***	0.964
Epilepsy duration	0.000	0.008	0.986	1.000
Febrile seizures	0.176	0.216	0.415	1.192
MRI lesion (+)	-0.147	0.137	0.283	0.864
Generalized epilepsy	-0.293	0.200	0.143	0.746
Infectious etiology	-1.093	0.452	**0.016***	0.335
Immune etiology	-0.699	0.331	**0.034***	0.497
Time since last seizure	0.253	0.083	**0.002***	1.288
Valproate	0.869	0.170	**0.000***	2.385
Antiseizure medications				
Levetiracetam	0.317	0.154	**0.039***	1.373
Topiramate	0.386	0.202	0.056	1.471
Lamotrigine	-0.119	0.210	0.573	0.888
Oxcarbazepine	0.587	0.207	**0.005***	1.799
Carbamazepine	0.816	0.233	**0.000***	2.262

Table S3. Associations between neutrophil-lymphocyte ratio and clinical parameters, including the antiseizure medications. Binary logistic regression was performed using tertile-based dichotomization. The dependent variable was dichotomized into the lowest tertile versus the combined higher tertiles.

	B	Standard error of coefficient	P value	Odds ratio
Sex	0.019	0.116	0.867	1.020
Age at sampling	-0.011	0.004	**0.003***	0.989
Epilepsy duration	0.004	0.007	0.545	1.004
Febrile seizures	0.490	0.198	**0.014***	1.632
MRI lesion (+)	0.070	0.119	0.558	1.072
Generalized epilepsy	-0.124	0.181	0.495	0.884
Infectious etiology	-0.256	0.374	0.495	0.775
Immune etiology	-0.335	0.285	0.240	0.715
Time since last seizure	0.384	0.073	**0.000***	1.468
Antiseizure medication				
Valproate	0.591	0.155	**0.000***	1.806
Levetiracetam	0.003	0.140	0.980	1.003
Topiramate	0.798	0.183	**0.000***	2.220
Lamotrigine	-0.076	0.197	0.699	0.927
Oxcarbazepine	-0.377	0.196	0.055	0.686
Carbamazepine	0.179	0.210	0.395	1.196

Table S4. Associations between platelet-albumin ratio and clinical parameters, including the antiseizure medications. Binary logistic regression was performed using tertile-based dichotomization. The dependent variable was dichotomized into the lowest tertile versus the combined higher tertiles.

	B	Standard error of coefficient	P value	Odds ratio
Sex	0.177	0.115	0.126	1.193
Age at sampling	0.004	0.003	0.239	1.004
Epilepsy duration	-0.006	0.007	0.409	0.995
Febrile seizures	0.081	0.207	0.694	1.085
MRI lesion (+)	0.030	0.118	0.801	1.030
Generalized epilepsy	-0.360	0.191	0.059	0.698
Infectious etiology	-0.673	0.390	0.085	0.510
Immune etiology	-0.494	0.283	0.081	0.610
Time since last seizure	0.283	0.072	**0.000***	1.327
Antiseizure medication				
Valproate	0.819	0.153	**0.000***	2.268
Levetiracetam	0.175	0.139	0.210	1.191
Topiramate	0.534	0.183	**0.004***	1.706
Lamotrigine	-0.233	0.202	0.251	0.792
Oxcarbazepine	-0.087	0.191	0.649	0.916
Carbamazepine	0.462	0.208	**0.026***	1.588

Table S5. Binary logistic regression analysis of valproate monotherapy user.

	VPA user vs. non-user (number or mean±SD)	p-value	Odds ratio (95% CI)
FAR	60.8±27.1 vs. 69.9±25.9	0.202	0.990 (0.976 to 1.005)
NLR	213.9±192.9 vs. 297.6±392.5	0.555	0.999 (0.996 to 1.002)
PLR	711.4±399.2 vs.1016.0±956.4	**0.002***	1.015 (1.005 to 1.025)
SII	424.9±349.7 vs. 687.2±901.1	**0.004***	0.984 (0.974 to 0.995)
Sex (female)	32/102 vs. 383/838	0.787	1.083 (0.609 to 1.926)
Onset age	28.5± 17.9 vs. 37.9±21.3	**0.028***	0.981 (0.964 to 0.998)
Epilepsy duration	6.2±8.4 vs.3.4±7.2	0.268	1.018 (0.986 to 1.051)
MRI lesion, yes	46/79 vs. 347/755	**0.004***	2.342 (1.311 to 4.181)
Focal epilepsy	71/102 vs. 661/838	0.405	2.342 (0.261 to 1.720)
Genetic etiology	23/102 vs. 117/838	0.910	0.669 (0.295 to 2.964)
Time since last seizure*	26/102, 47/102, 29/102 vs. 379/812, 245/812, 188/812	0.024*	0.935 (1.056 to 2.139)
Platelets	208.7±59.2 vs. 239.1±61.8	**0.000***	0.971 (0.958 to 0.985)
Lymphocytes	33.7±11.1 vs. 29.7±10.9	0.158	1.045 (0.983 to 1.112)
Neutrophils	54.9±12.0 vs. 60.5±12.5	0.576	1.000 (0.999 to 1.001)
White blood cells	6.5±2.1 vs. 7.1±2.8	0.816	0.946 (0.590 to 1.515)

* Time since last seizure was categorized <1 week, 1 week ~1 month and ≧1 month

VPA, valproic acid; FAR, fibrinogen-albumin ratio; NLR, neutrophil-lymphocyte ratio; PLR, platelet-lymphocyte ratio; SII, systemic inflammatory index; SD, standard deviation; CI, confidence interval

Table S6. Binary logistic regression analysis of topiramate monotherapy user

	TPM user vs non-user (number or mean±SD)	p-value	Odds ratio (95% CI)
FAR	63.1±13.4 vs. 69.9±25.9	0.930	0.999 (0.977 to 1.022)
NLR	233.9±218.4 vs. 297.6±392.5	0.576	1.001 (0.998 to 1.004)
PLR	853.6±396.2 vs.1016.0±956.4	0.966	1.000 (0.993 to 1.006)
SII	533.5±379.7 vs. 687.2±901.1	0.895	0.999 (0.992 to 1.007)
Onset age	20.6±13.4 vs. 37.9±21.3	**0.005***	0.955 (0.925 to 0.986)
Epilepsy duration	9.6±12.0 vs. 3.4±7.2	0.085	1.033 (0.996 to 1.071)

TPM, topiramate

Table S7. Binary logistic regression analysis of carbamazepine monotherapy user

	CBZ user vs non-user (number or mean±SD)	p-value	Odds ratio (95% CI)
FAR	61.6±20.9 vs. 69.9±25.9	0.285	0.988 (0.968 to 1.010)
NLR	197.8±136.6 vs. 297.6±392.5	0.978	1.000 (0.995 to 1.005)
PLR	778.9±387.8 vs. 1016.0±956.4	0.586	1.002 (0.995 to 1.009)
SII	498.0±353.8 vs.687.2±901.1	0.507	0.997 (0.986 to 1.007)
Onset age	23.7±16.8 vs. 37.9±21.3	0.438	0.991 (0.970 to 1.013)
Epilepsy duration	13.5±12.7 vs. 3.4±7.2	**0.000***	1.078 (1.046 to 1.110)
Time since last seizure†	14/54, 24/54, 16/54 vs. 379/812, 245/812, 188/812	0.462	1.169 (0.772 to 1.769)
Lymphocytes	33.0±8.7 vs. 29.7±10.9	0.613	0.979 (0.901 to 1.063)
Neutrophils	55.8±9.8 vs. 60.5±12.5	0.885	1.001 (0.666 to 1.001)
White blood cells	6.1±2.2 vs. 7.1±2.85	0.798	0.896 (0.387 to 2.073)

† Time since last seizure was categorized <1 week, 1 week ~1 month and ≧1 month

CBZ, carbamazepine

Table S8. Binary logistic regression analysis of lamotrigine monotherapy user

	LTG user vs non-user (number or mean±SD)	p-value	Odds ratio (95% CI)
FAR	63.8±13.5 vs. 69.9±25.9	0.304	1.008(0.993 to1.023)
NLR	181.0±99.3 vs. 297.6±392.5	0.083	0.982(0.961 to 1.002)
PLR	796.0±386.8 vs. 1016.0±956.4	0.050	0.991(0.982 to 1.000)
SII	466.8±321.9 vs. 687.2±901.1	0.055	1.014(1.000 to 1.029)
Onset age	24.1±17.4 vs. 37.9±21.3	**0.002***	0.963(0.939 to 0.986)
Epilepsy duration	7.1±6.5 vs. 3.4±7.2	0.590	1.010(0.973 to 1.049)
Focal epilepsy	33/51 vs. 661/838	0.995	1.002(0.491 to 2.046)
Time since last seizure*	9/51, 23/51, 19/51 vs. 379/812, 245/812, 188/812	**0.006***	1.801(1.184 to 2.738)
Neutrophils	55.4±8.9 vs. 60.5±12.5	0.476	0.960(0.857 to 1.074)
Lymphocytes	34.1±7.7 vs. 29.7±.9	0.243	0.909(0.775 to 1.067)
White blood cells	6.5±1.8 vs. 7.1±2.8	0.688	1.036(0.872 to 1.231)

† Time since last seizure was categorized <1 week, 1 week ~1 month and ≧1 month

LTG, lamotrigine

Table S9. Binary logistic regression analysis of levetiracetam monotherapy user

	LEV user vs non-user (number or mean±SD)	p-value	Odds ratio (95% CI)
FAR	64.2±25.0 vs. 69.9±25.9	0.878	1.001 (0.991 to 1.010)
NLR	259.5±312.4 vs. 297.6±392.5	0.083	0.998 (0.995 to 1.000)
PLR	997.5±1106.5 vs. 1016.0±956.4	**0.039***	0.997 (0.993 to 1.000)
SII	667.6±1044.3 vs. 687.2±901.1	**0.029***	1.004 (1.000 to 1.008)
Onset age	29.8±16.9 vs. 37.9±21.3	**0.000***	0.977 (0.999 to 0.988)
Time since last seizure*	36/155, 56/155, 63/155 vs. 379/812, 245/812, 188/812	**0.000***	1.638 (1.276 to 2.103)

† Time since last seizure was categorized <1 week, 1 week ~1 month and ≧1 month

LEV, levetiracetam

Table S10. Multiple linear regression models for each inflammatory index in monotherapy population.

	Standardized beta coefficient (p value)			
	FAR	NLR	PLR	SII
Sex	-0.070(**0.019***)	-	-	-
Onset age	0.361(**<0.001***)	0.048(0.145)	-0.009(0.782)	0.000(0.988)
Epilepsy duration	0.100(**0.003***)	-0.034(0.290)	-0.048(0.141)	-0.053(0.081)
Febrile seizure	-0.010(0.747)	-0.038(0.190)	-0.033(0.255)	-0.035(0.204)
Time since last seizure*	-0.113(**<0.001***)	-0.177(**<0.001***)	-0.162(**<0.001***)	-0.154(**<0.001***)
Seizure frequency	-0.033(0.275)	-	-0.075(**0.011***)	-
MRI lesion	0.044(0.232)	0.045(0.130)	-	-
Generalized epilepsy	0.083(**0.009***)	-0.017(0.566)	-0.028(0.365)	-0.028(0.331)
Structural etiology	0.043(0.245)	-	-	-
Infectious etiology	0.257(**<0.001***)	0.196(**<0.001***)	0.179(**<0.001***)	0.177(**<0.001***)
Valproate	0.015(0.722)	-0.015(0.636)	-0.069(**0.038***)	-0.055(0.074)
Levetiracetam	0.032(0.492)	-	-	-
Topiramate	-	-	-	-
Lamotrigine	0.041(0.271)	-0.025(0.409)	-0.024(0.429)	-0.021(0.462)
Oxcarbazepine	0.004(0.916)	-0.001(0.978)	-0.036(0.248)	-0.022(0.463)

Carbamazepine	-0.017(0.660)	-0.014(0.652)	-0.025(0.422)	-0.028(0.348)
Drug-naïve	0.077(0.209)	0.024(0.524)	-0.014(0.715)	0.001(0.982)
